# Defective Ti_2_Nb_10_O_27.1_: an advanced anode material for lithium-ion batteries

**DOI:** 10.1038/srep17836

**Published:** 2015-12-03

**Authors:** Chunfu Lin, Shu Yu, Hua Zhao, Shunqing Wu, Guizhen Wang, Lei Yu, Yanfang Li, Zi-Zhong Zhu, Jianbao Li, Shiwei Lin

**Affiliations:** 1 Key Laboratory of Ministry of Education for Advanced Materials in Tropical Island Resources, College of Materials and Chemical Engineering, Hainan University, Haikou 570228, PR China; 2 Department of Physics and Institute of Theoretical Physics and Astrophysics, Xiamen University, Xiamen 361005, PR China

## Abstract

To explore anode materials with large capacities and high rate performances for the lithium-ion batteries of electric vehicles, defective Ti_2_Nb_10_O_27.1_ has been prepared through a facile solid-state reaction in argon. X-ray diffractions combined with Rietveld refinements indicate that Ti_2_Nb_10_O_27.1_ has the same crystal structure with stoichiometric Ti_2_Nb_10_O_29_ (Wadsley-Roth shear structure with *A2/m* space group) but larger lattice parameters and 6.6% O^2–^ vacancies (*vs.* all O^2–^ ions). The electronic conductivity and Li^+^ion diffusion coefficient of Ti_2_Nb_10_O_27.1_ are at least six orders of magnitude and ~2.5 times larger than those of Ti_2_Nb_10_O_29_, respectively. First-principles calculations reveal that the significantly enhanced electronic conductivity is attributed to the formation of impurity bands in Ti_2_Nb_10_O_29–*x*_ and its conductor characteristic. As a result of the improvements in the electronic and ionic conductivities, Ti_2_Nb_10_O_27.1_ exhibits not only a large initial discharge capacity of 329 mAh g^–1^ and charge capacity of 286 mAh g^–1^ at 0.1 C but also an outstanding rate performance and cyclability. At 5 C, its charge capacity remains 180 mAh g^–1^ with large capacity retention of 91.0% after 100 cycles, whereas those of Ti_2_Nb_10_O_29_ are only 90 mAh g^–1^ and 74.7%.

The great success of lithium-ion batteries (LIBs) in portable electronic devices has triggered considerable efforts for their further applications in electric vehicles (EVs)^1^. The two key requirements of the LIBs for EVs are high power density and high energy density, which respectively determine how fast and far the EVs can travel on a single charge. Unfortunately, the commonly used graphite anode material suffers from its poor rate performance and safety in spite of its large theoretical capacity (372 mAh g^–1^)^2^. In this regard, many other anode materials (such as intercalation-type TiO_2_ and Li_4_Ti_5_O_12_, alloying-type Si and Sn, and conversion-type Fe_2_O_3_ and Co_3_O_4_) have been developed. Among them, Li_4_Ti_5_O_12_ probably has received the most attention^3^. Li_4_Ti_5_O_12_ shows a spinel crystal structure with its cations located at both octahedral and tetrahedral sites^4^. It exhibits a high working potential (~1.57 V *vs.* Li/Li^+^) and negligible volume change (<0.1%) during the lithiation and delithiation processes, respectively lead to its high safety and good cyclability. Through the modifications by doping with foreign ions, compositing a second conductive phase and/or reducing the particle size, its intrinsically low electronic conductivity and Li^+^ ion diffusion coefficient can be significantly improved, resulting in its high rate performance^5^,^6^. However, it has a small theoretical capacity of only 175 mAh g^–1^ between 3.0 and 1.0 V *vs.* Li/Li^+^, which cannot be effectively increased. Thus, Li_4_Ti_5_O_12_ cannot fulfil the requirement of high energy density for EVs although it can fulfil that of high power density.

As a new type of anode materials with large theoretical capacities, Ti–Nb–O compounds have attracted great research interest very recently. Two known Ti–Nb–O anode materials, TiNb_2_O_7_ and Ti_2_Nb_10_O_29_, were firstly reported by Han *et al.* and Wu *et al.* in 2011 and 2012, respectively^7^,^8^,^9^. In these compounds, there is a one-electron transfer between Ti^4+^ and Ti^3+^ ions and two-electron transfer between Nb^5+^ and Nb^3+^ ions. As a result, TiNb_2_O_7_ has a large theoretical capacity of 388 mAh g^–1^ based on the five-electron transfer per formula unit, and that of Ti_2_Nb_10_O_29_ is 396 mAh g^–1^ based on its 22-electron transfer. These theoretical capacities are ~1.2 times larger than that of Li_4_Ti_5_O_12_ and even surpass that of graphite. TiNb_2_O_7_ and Ti_2_Nb_10_O_29_ exhibit Wadsley-Roth shear structures constructed by *m* × *n* × ∞ (*m* = *n* = 3 for TiNb_2_O_7_, Fig. 1a; *m* = 4 and *n* = 3 for Ti_2_Nb_10_O_29_, Fig. 1b) ReO_3_-type blocks, where *m* and *n* are respectively the length and width of the blocks in numbers of octahedral^10^. All cations (Ti^4+^ and Nb^5+^ ions with molar ratios of 1 : 2 for TiNb_2_O_7_ and 1 : 5 for Ti_2_Nb_10_O_29_) randomly occupy the octahedral sites connected by edges and corners. Since no cations are resided at the tetrahedral sites, the Wadsley-Roth shear structure is more open than the spinel structure, inferring its larger Li^+^ ion diffusion coefficient. In spite of the above advantages, TiNb_2_O_7_ and Ti_2_Nb_10_O_29_ suffer from their intrinsically low electronic conductivities and Li^+^ ion diffusion coefficients, which significantly limit their rate performances.

To fulfil the requirement of high power density, it is highly necessary to modify TiNb_2_O_7_ and Ti_2_Nb_10_O_29_. However, very limited studies have followed the reports from Han *et al.* and Wu *et al.* so far^11^,^12^,^13^,^14^,^15^,^16^,^17^. Only TiNb_2_O_7_ nanoparticles, nanofibers and nanoporous particles were prepared and exhibited good rate performances due to the short transport distance for electrons and Li^+^ ions within the TiNb_2_O_7_ particles^11^,^12^,^13^,^14^,^15^. Nevertheless, their fabrications are rather complex and their tap densities are rather small, limiting their practical applications. Crystal structure modification (including doping) has been demonstrated as an effective and facile strategy to improve the rate performance of intercalation-type electrode materials due to the resultant improvements of the electronic conductivity and/or Li^+^ ion diffusion coefficient^5^,^6^. In this study, for the first time, we have employed the strategy of crystal structure modification to improve the rate performance of Ti_2_Nb_10_O_29_. Using a facile solid-state reaction method, defective Ti_2_Nb_10_O_29–*x*_ containing O^2–^ vacancies has been successfully fabricated. Its crystal structure, material properties and electrochemical performances has been intensively studied and compared with the stoichiometric Ti_2_Nb_10_O_29_. The results based on the experiments, Rietveld refinements and first-principle calculations reveal that Ti_2_Nb_10_O_29–*x*_ has a larger unit cell volume, enhanced electronic conductivity, improved Li^+^ ion diffusion coefficient, large capacity, high rate performance and good cyclability. Therefore, Ti_2_Nb_10_O_29–*x*_ is able to fulfil the two key requirements of high power density and high energy density for EVs.

## Results and Discussion

### Crystal structure analysis

The observed, calculated, error XRD patterns for the two prepared Ti–Nb–O samples are plotted in Fig. 2. The sharp diffraction peaks are indicative of their good crystallinity rooted in the high-temperature calcination at 1200 °C. The pattern of the Ti–Nb–O sample calcined in air (Fig. 2a) matches well with a Wadsley-Roth shear structure (*A2/m* space group) and all peaks are in good agreement with those of JCPDS card No. 72-0159, which suggest the formation of pure Ti_2_Nb_10_O_29_. The peaks of the Ti–Nb–O sample calcined in argon (Fig. 2b) are very similar to those of Ti_2_Nb_10_O_29_, suggesting the two samples share the same basic crystal structure. No diffractions from TiO_2_, Nb_2_O_5_, TiNb_2_O_7_ or TiNb_24_O_62_ can be observed, indicating that the employment of the argon atmosphere did not influence the formation of Ti_2_Nb_10_O_29_-type crystals. A previous study confirmed that a similar reaction at non-oxidizing atmosphere resulted in the generation of O^2–^ vacancies and cations in lower valence state and thus the formation of nonstoichiometric transition oxide^18^. Hence, it can be deduced that Ti_2_Nb_10_O_29–*x*_ is a reasonable chemical formula to represent the Ti–Nb–O sample calcined in argon, in which Ti^3+^ and Nb^4+^ ions exist together with O^2–^ vacancies. Using the crystal data of Ti_2_Nb_10_O_29_ as the initial crystal data for Ti_2_Nb_10_O_29–*x*_, the spectrum of Ti_2_Nb_10_O_29–*x*_ was successfully refined by the Rietveld method. As a comparison, the refinement of Ti_2_Nb_10_O_29_ was also carried on. Their Rietveld refinement results are summarized Table 1. As can be seen, Ti_2_Nb_10_O_29–*x*_ exhibits a considerably large amount of O^2–^ vacancies (6.6% *vs.* all O^2–^ ions) and thus a chemical formula of Ti_2_Nb_10_O_27.1_. In addition, it has larger lattice parameters and a larger unit cell volume than those of the stoichiometric Ti_2_Nb_10_O_29_. These increases can be due to the existence of the O^2–^ vacancies, Ti^3+^ ions with a larger size (0.67 Å) than that of Ti^4+^ ions (0.605 Å) and Nb^4+^ ions with a larger size (0.68 Å) than that of Nb^5+^ ions (0.64 Å)^19^.

**Figure 1:**
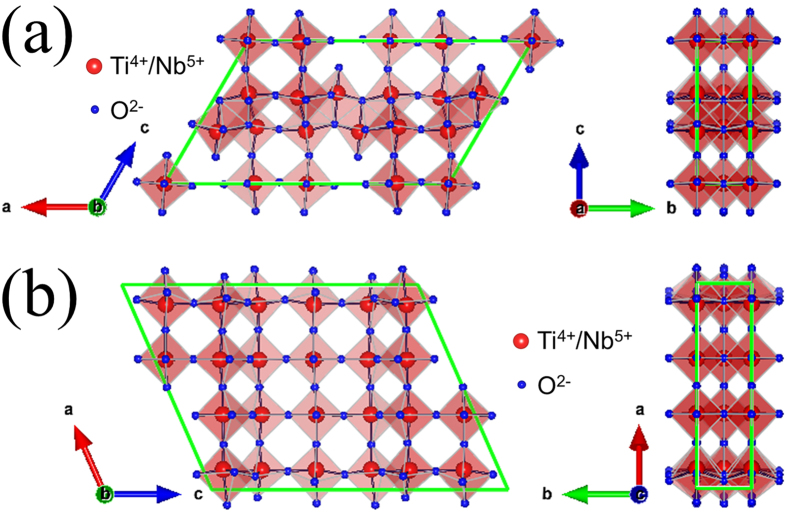
Schematic representations of the unit cells for (**a**) TiNb_2_O_7_ and (**b**) Ti_2_Nb_10_O_29_.

**Figure 2:**
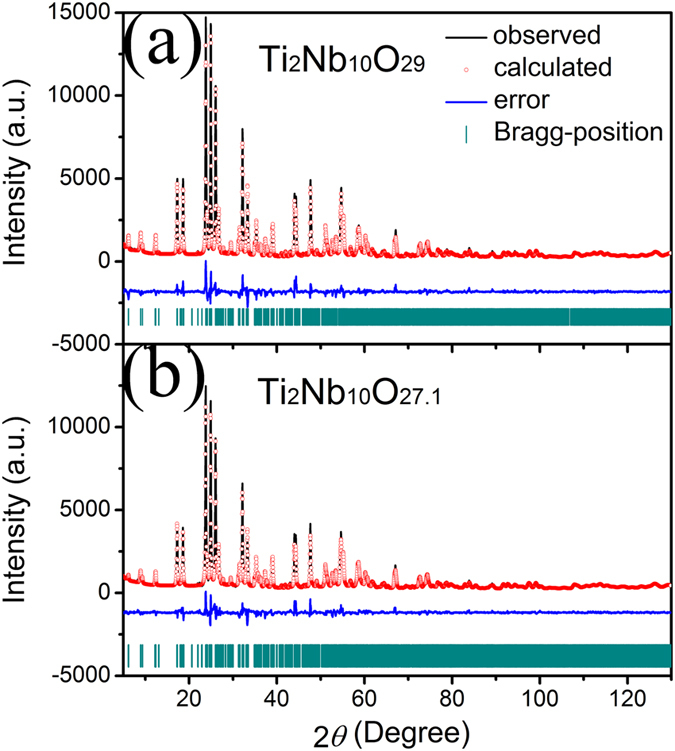
XRD patterns and Rietveld refinement results of Ti_2_Nb_10_O_29_ and Ti_2_Nb_10_O_27.1_.

**Figure 3:**
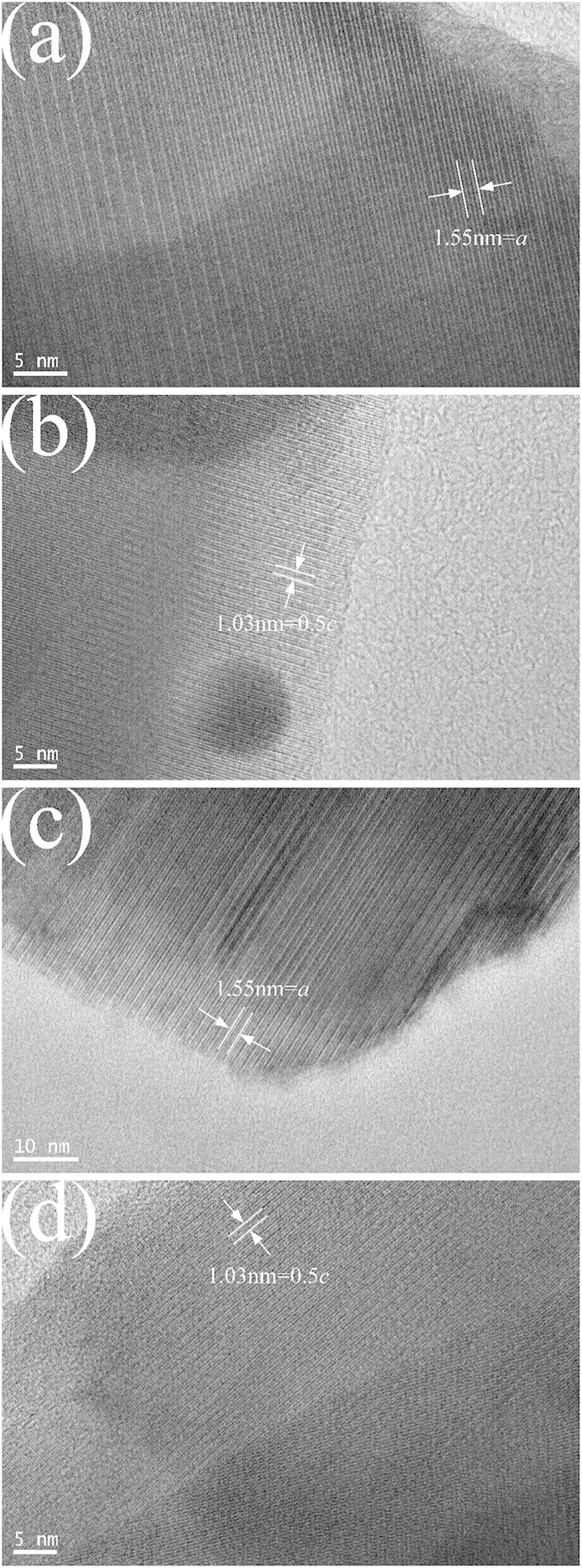
HRTEM images of (**a,b**) Ti_2_Nb_10_O_29_ and (**c, d**) Ti_2_Nb_10_O_27.1_.

The crystalline characteristics of Ti_2_Nb_10_O_29_ and Ti_2_Nb_10_O_27.1_ were further examined by the HRTEM tests (Fig. 3). As can be seen in Fig. 3a,b, the atomic layers in Ti_2_Nb_10_O_29_ are positioned in orderly and repeated patterns. When combining the three white stripes indicated by the arrows as a unit in Fig. 3a, the layers can be considered as an equidistant arrangement of the unit. The interval between the units is 1.03 nm, which is equal to a half of the lattice parameter *c* (Table 1). As shown in Fig. 1b, *c* is parallel to the width direction of the block (*i.e.*, *n* direction). Thus, the unit can reflect the width characteristic of the block. Similarly, in Fig. 3b, the interval between the units is 1.55 nm, corresponding to the lattice parameter *a*. This unit can reflect the length and width characteristics of the block since *a* is not parallel to or perpendicular to the length/width direction of the block (*i.e.*, *m*/*n* direction). When comparing Fig. 3a,c as well as Fig. 3b,d, it can be found that Ti_2_Nb_10_O_27.1_ and Ti_2_Nb_10_O_29_ have the same units, which indicates that the two samples have the same block structure and thus similar crystal structures. This finding is in good agreement with the XRD result.

### Particle morphology and size

**Figure 4:**
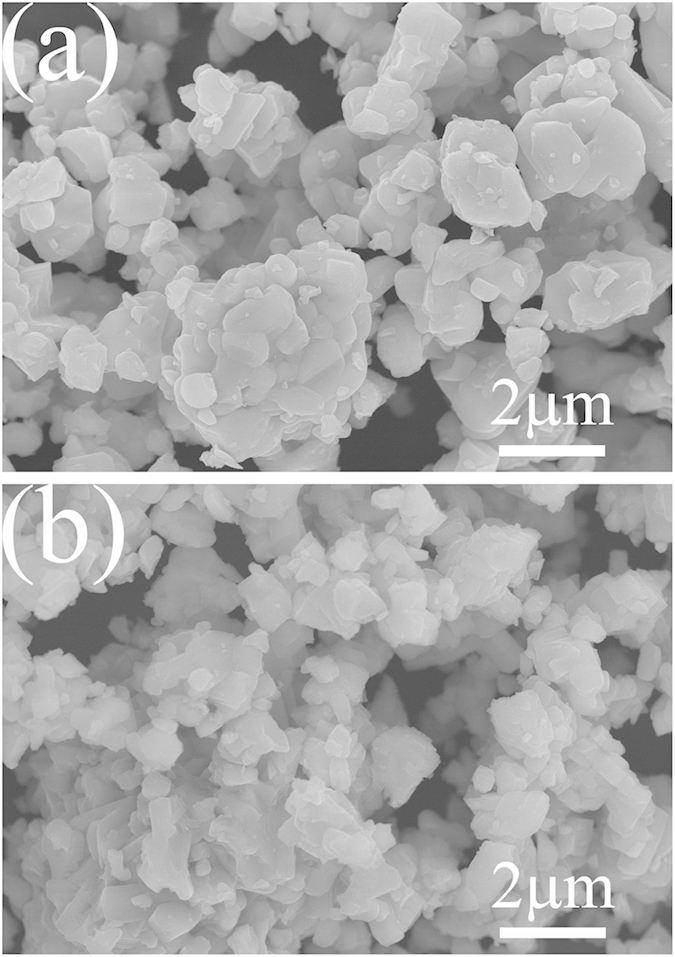
FESEM images of (**a**) Ti_2_Nb_10_O_29_ and (**b**) Ti_2_Nb_10_O_27.1_.

Figure 4a,b illustrate the particle morphologies and sizes of Ti_2_Nb_10_O_29_ and Ti_2_Nb_10_O_27.1_, respectively. Both samples exhibit similar morphologies with wide primary particle-size distributions ranging from less than 100 nm to more than 1 μm. Aggregates exist in both samples. Therefore, the morphologies show the common features of the powders from solid-state reaction. The BET specific surface area of Ti_2_Nb_10_O_27.1_ is 1.30 m^2^ g^–1^, which is very similar to that of its stoichiometric counterpart (1.26 m^2^ g^–1^). This comparison suggests that the different calcination atmosphere negligibly affects the particle size.

### Electronic conductivity

The electronic conductivities of Ti_2_Nb_10_O_29_ and Ti_2_Nb_10_O_27.1_ were determined based on a two-probe direct current method. Ti_2_Nb_10_O_29_ has an electronic conductivity which is so low that it cannot be accurately measured using the electrochemical workstation. Since the electrochemical workstation has a current limit of 1 nA, it can be deduced that its electronic conductivity is below 1 × 10^–9^ S cm^–1^, inferring its insulator characteristic. This result can be due to the fact that there are no free electrons within its cations (Ti^4+^ and Nb^5+^ ions) in their highest valence states. In sharp contrast, the electronic conductivity of Ti_2_Nb_10_O_27.1_ is increased by at least six orders of magnitude to a large value of 1.06 × 10^–3^ S cm^–1^. Ti_2_Nb_10_O_27.1_ presents considerable amounts of Ti^3+^ and Nb^4+^ ions with unpaired electrons, which can greatly contribute to the significant improvement of the electronic conductivity^20^. It is known that the electronic conductivities of doped metal oxides are generally in the range of only 10^–9^–10^–7 ^S cm^–1^^5^,^6^. For instance, the values of Li_3.9_Ni_0.15_Ti_4.95_O_12_, Li_3.8_Cu_0.3_Ti_4.9_O_12_ and Li_3.9_Cr_0.3_Ti_4.8_O_12_ are 3.1 × 10^–8^, 3.6 × 10^–8^ and 4.7 × 10^–8^ S cm^–1^, respectively^5^,^6^. Therefore, compared with doping, the crystal structure modification based on the production of O^2–^ vacancies in this work is a much more effective strategy to enhance the electronic conductivity of metal oxides.

**Figure 5:**
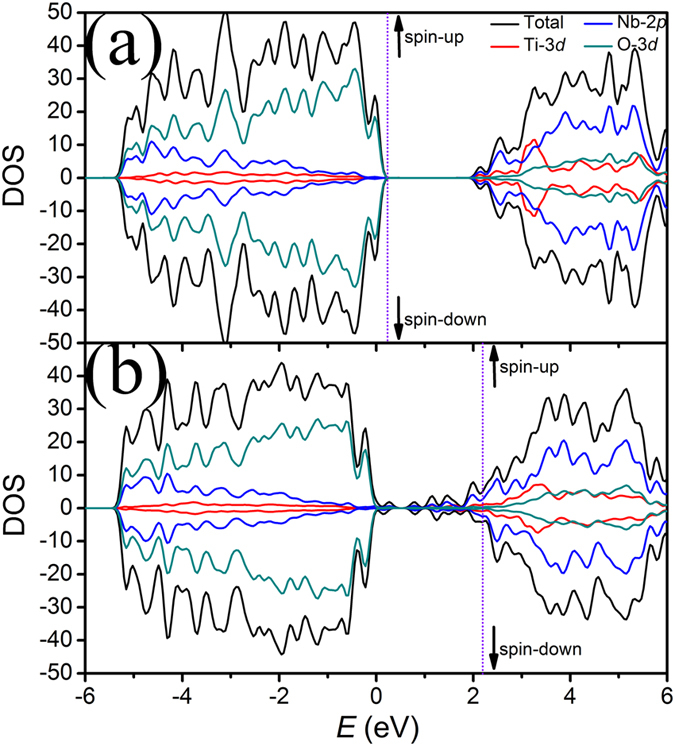
Calculated TDOS and PDOS of (**a**) Ti_2_Nb_10_O_29_ (Ti_4_Nb_20_O_58_) and (**b**) Ti_2_Nb_10_O_27_ (Ti_4_Nb_20_O_54_). Fermi levels are shown by dot lines.

In order to understand more about the physical essence of the pristine Ti_2_Nb_10_O_29_ and its defective material, DFT calculations were carried out, and their calculated total density of states (TDOS) and partial density of states (PDOS) are illustrated in Fig. 5. As can be seen in Fig. 5a, for the valence band of Ti_2_Nb_10_O_29_, O 2*p*, Ti 3*d* and Nb 4*d* states fill up the relative lower electronic states from −5.4 eV to 0.3 eV. The clear overlaps of these states indicate significant hybridization between Ti 3*d* and O 2*p* orbitals and between Nb 4*d* and O 2*p* orbitals (*i.e.*, ionic Ti–O and Nb–O bonds are formed.). The conduction band mainly comprises unoccupied Ti and Nb states, located from 1.9 eV to 6 eV. The resultant band gap is ~1.6 eV, which is large enough to confirm the insulator characteristic of Ti_2_Nb_2_O_29_ since it is well known that the standard DFT calculation underestimates the band gap. Therefore, the electronic conductivity of the pristine Ti_2_Nb_10_O_29_ is very low. In contrast, as shown in Fig. 5b, the Fermi level in Ti_2_Nb_10_O_27_ is inside of some bands, suggesting that Ti_2_Nb_10_O_27_ is no longer an insulator but changed to be a conductor. In addition, impurity bands appear close to but below the Fermi level. These impurity bands are composed of the Ti *3d* and Nb 4*d* states with slightly hybridization with the O 2*p* states. After the calcination in argon, the production of O^2–^ vacancies in the defective material alters the electron configurations of Ti and Nb ions, leading to the formation of the impurity bands, the conductor characteristic and thus the huge improvement of the electronic conductivity.

### Li^+^ ion diffusion coefficient

**Figure 6:**
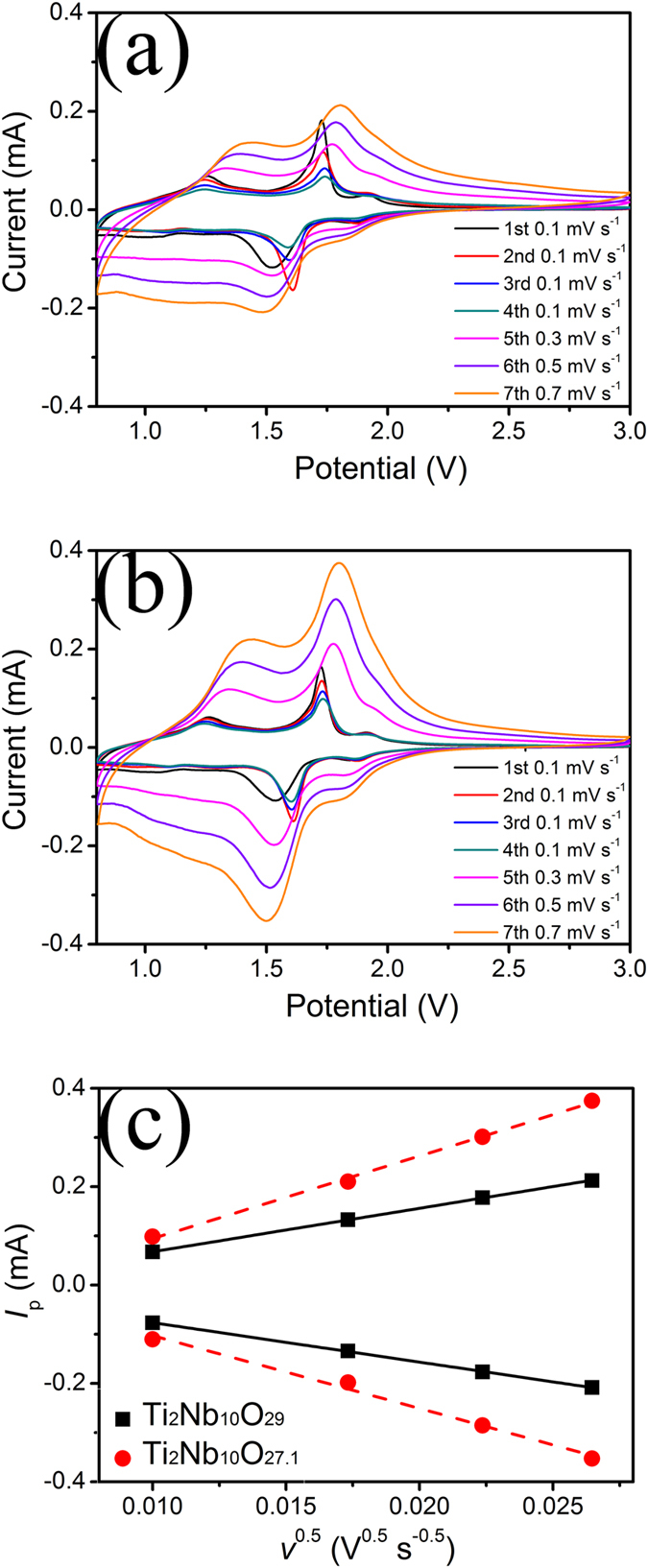
CV curves of (**a**) Ti_2_Nb_10_O_29_/Li and (**b**) Ti_2_Nb_10_O_27.1_/Li cells, and (**c**) relationship between peak current of cathodic/anodic reaction *I*_p_ and square root of sweep rate *v*^0.5^.

The Li^+^ diffusion coefficients of Ti_2_Nb_10_O_29_ and Ti_2_Nb_10_O_27.1_ were determined using the CV technique. The Ti_2_Nb_10_O_29_/Li and Ti_2_Nb_10_O_27.1_/Li cells were tested at a scanning rate of 0.1 mV s^–1^ for four cycles and then successively at 0.3, 0.5 and 0.7 mV s^–1^ for one cycle each between 3.0 and 0.8 V *vs.* Li/Li^+^ at room temperature, as displayed in Fig. 6a,b. For each cell at 0.1 mV s^–1^, the intensive cathodic peak shifts to a larger potential after the first cycle. This shift may be attributed to the variation of the electronic structure of Ti_2_Nb_10_O_29_/Ti_2_Nb_10_O_27.1_ rooted in the irreversible lithiation process in the first cycle (note that the initial Coulombic efficiency for each cell is not 100% as presented below)^12^,^17^. In fact, such shift can also be observed in other intercalation-type anode materials, such as Li_4_Ti_5_O_12_^21^. Each cycle of the Ti_2_Nb_10_O_29_/Li cell shows two cathodic peaks and three anodic peaks, and that of the Ti_2_Nb_10_O_27.1_/Li cell also exhibits five peaks at the similar positions but with larger intensities. These peaks can be ascribed to the Ti^3+^/Ti^4+^, Nb^4+^/Nb^5+^ and Nb^3+^/Nb^4+^ redox couples. A previous report reveals that the Ti^4+^ and Nb^5+^ ions in TiNb_2_O_7_ are simultaneously and continuously reduced during the discharge (lithiation) process^15^. Thus, each of the five peaks may be assigned to two or more redox couples. For instance, the cathodic peaks centered at ~1.88 V *vs.* Li/Li^+^ (at 0.1 mV s^–1^) may correspond to Ti^3+^/Ti^4+^ and Nb^4+^/Nb^5+^ redox couples. Among all the peaks at 0.1 mV s^–1^, the cathodic one centered at ~1.61 V *vs.* Li/Li^+^ and the anodic one centered at ~1.73 V *vs.* Li/Li^+^ are relatively intensive. Taking the middle points between these two peaks, The average working potentials of both cells were determined to be ~1.71 V *vs.* Li/Li^+^, which is similar to those of the TiNb_2_O_7_/Li cell (~1.64 V *vs.* Li/Li^+^)^17^ and the Li_4_Ti_5_O_12_/Li cell (~1.57 V *vs.* Li/Li^+^). Such reasonably high working potentials are able to suppress the reduction of electrolyte and avoid the formation of thick solid-electrolyte interphase (SEI) layers and lithium dendrites on Ti_2_Nb_10_O_27.1_ and Ti_2_Nb_10_O_29_ particle surfaces, resulting in their high safety for EVs. The potential differences between the cathodic and anodic peaks reflect the polarization degree of the cells. The Ti_2_Nb_10_O_29_/Li cell shows a polarization of 0.123 V based on the intensive cathodic and anodic peaks at 0.1 mV s^–1^, while that of the Ti_2_Nb_10_O_27.1_/Li cell is lowered to 0.113 V. This smaller polarization of the Ti_2_Nb_10_O_27.1_/Li cell together with its sharper cathodic and anodic peaks is indicative of its better electrochemical kinetics.

In addition, there is a linear relationship between the peak current of the intensive cathodic/anodic reaction *I*_p_ and the square root of the sweep rate *v*^0.5^, as illustrated in Fig. 6c. As a result, the Li^+^ ion diffusion coefficient *D* can be calculated based on the Randles–Sevcik equation^22^:


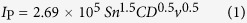


where *S*, *n*, and *C* are respectively the electrode surface area of contact between the active materials and electrolyte, the charge transfer number and the molar concentration of Li^+^ ions in solid. Ti_2_Nb_10_O_29_ has Li^+^ ion diffusion coefficients of 5.43 × 10^–15^ and 6.52 × 10^–15^ cm^2^ s^–1^ during the lithiation and delithiation processes, respectively. In contrast, the corresponding values of Ti_2_Nb_10_O_27.1_ are increased to 1.84 × 10^–14^ and 2.37 × 10^–14^ cm^2^ s^–1^. Since the Li^+^ ion diffusion coefficients for Ti_2_Nb_10_O_27.1_ and Ti_2_Nb_10_O_29_ during the lithiation process are smaller than those during the delithiation process, it can be confirmed that the lithiation process is the rate-limiting step for both samples. Obviously, compared with Ti_2_Nb_10_O_29_, Ti_2_Nb_10_O_27.1_ delivers a ~2.5 times larger Li^+^ ion diffusion coefficient during the lithiation process. The increases in the Li^+^ ion diffusion coefficient may be attributed to the crystalline characteristics of Ti_2_Nb_10_O_27.1_. Its larger unit cell volume may lead to wider Li^+^ ion transport paths in its crystal structure, and its 6.6% O^2–^ vacancies may imply more Li^+^ ion transport paths. Both improvements can facilitate the transport of Li^+^ ion in the active particles during the electrochemical reaction, resulting in its larger Li^+^ ion diffusion coefficients. Moreover, it is worth noting that the average Li^+^ ion diffusion coefficient of Ti_2_Nb_10_O_27.1_ is two orders of magnitude larger than that of Li_4_Ti_5_O_12_ (1.81 × 10^–16^ cm^2^ s^–1^), further confirming the advanced crystal structure of the defective Ti_2_Nb_10_O_27.1_.

### Discharge–charge performance

**Figure 7:**
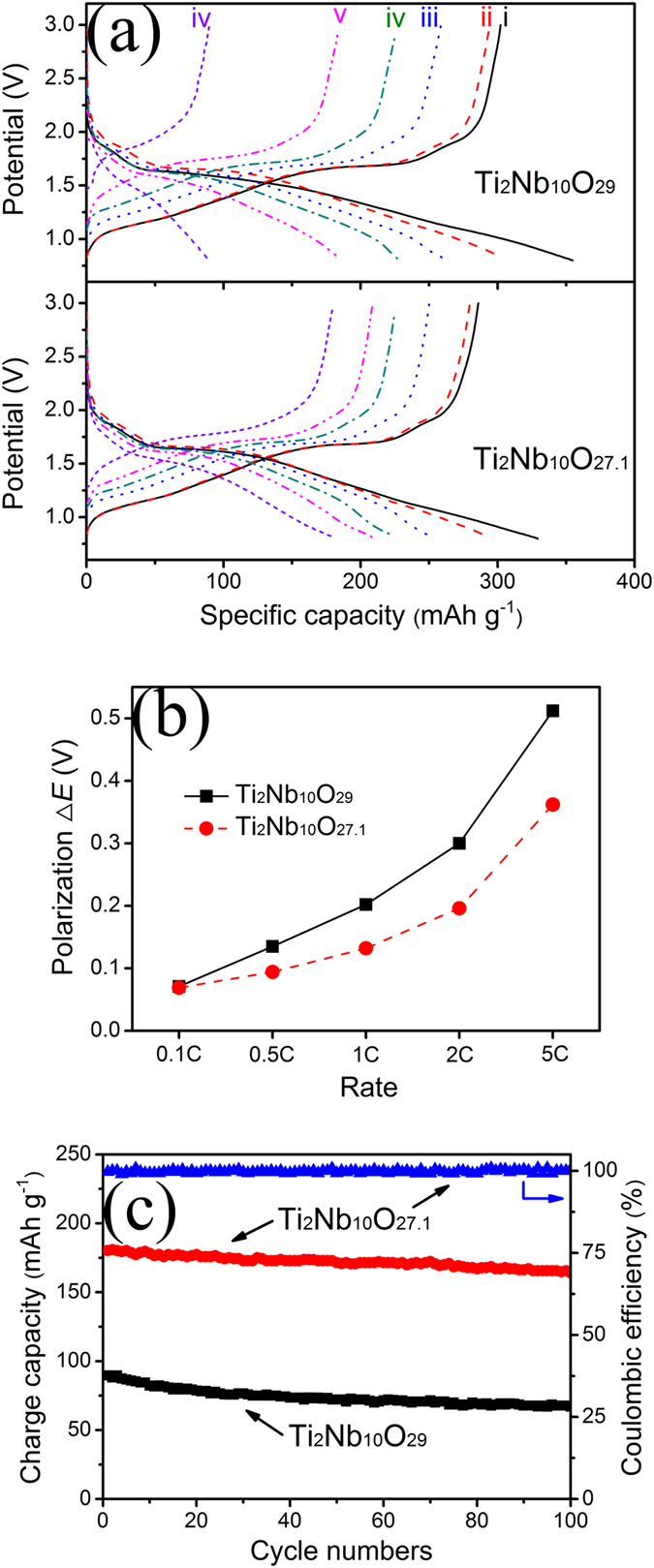
(**a**) (i) Initial discharge–charge curves at 0.1 C, second discharge–charge curves at (ii) 0.1 C, (iii) 0.5 C, (iv) 1 C, (v) 2 C and (vi) 5 C of Ti_2_Nb_10_O_29_/Li and Ti_2_Nb_10_O_27.1_/Li cells; (b) polarization Δ*E* of Ti_2_Nb_10_O_29_/Li and Ti_2_Nb_10_O_27.1_/Li cells at 0.1, 0.5, 1, 2 and 5 C; and (c) cyclability of Ti_2_Nb_10_O_29_/Li and Ti_2_Nb_10_O_27.1_/Li cells at 5 C along with Coulombic efficiency of Ti_2_Nb_10_O_27.1_/Li cell. Identical discharge–charge rates were used.

The discharge–charge behaviours of the Ti_2_Nb_10_O_29_/Li and Ti_2_Nb_10_O_27.1_/Li cells were examined under galvanostatic conditions by performing several discharge–charge cycles. Figure 7a compares their discharge–charge curves at different rates (0.1, 0.5, 1, 2 and 5 C) between 3.0 and 0.8 V *vs.* Li/Li^+^. Despite of the differences in the reversible capacities, the curve-shapes for the two cells are similar, demonstrating the defects (O^2–^ vacancies, Ti^3+^ ions and Nb^4+^ ions) in Ti_2_Nb_10_O_27.1_ do not affect the fundamental electrochemical reaction mechanism. At 0.1 C, each sample shows a short discharge plateau at ~1.65 V *vs.* Li/Li^+^ and a short charge plateau at ~1.68 V *vs.* Li/Li^+^, which match well with the two intensive peaks in the CV curves (Fig. 6). These two plateaus can correspond to a two-phase reaction^9^. The sloping curves before and after the plateau region are indicative of two different solid-solution reactions. During the first cycle at 0.1 C, the Ti_2_Nb_10_O_29_/Li cell delivers a discharge capacity of 354 mAh g^–1^. At the same rate, the corresponding capacity for the Ti_2_Nb_10_O_27.1_/Li cell is slightly decreased to 329 mAh g^–1^. It is known that all the electrochemical energy in Ti_2_Nb_10_O_29_ comes from the reversible redox reactions between Ti^3+^ and Ti^4+^ ions, between Nb^4+^ and Nb^5+^ ions, and between Nb^3+^ and Nb^4+^ ions. Since there are considerable amounts of Ti^3+^ and Nb^4+^ ions in the defective Ti_2_Nb_10_O_27.1_, its contents of Ti^4+^ and Nb^5+^ ions are smaller than those in its stoichiometric counterpart, leading to its lower initial discharge capacity. However, the Ti_2_Nb_10_O_27.1_/Li cell exhibits a larger initial Coulumbic efficiency (86.9%) than that of its stoichiometric counterpart (82.8%) probably due to its better electrochemical kinetics. Thus, its initial charge capacity (286 mAh g^–1^) can approach that of its stoichiometric counterpart (294 mAh g^–1^).

### Rate performance and power density

With increasing the rate, the plateaus become inconspicuous; the discharge curves monotonically drop and the corresponding charge curves monotonically rise, indicating the increasing polarization. The polarization Δ*E vs.* rate of both cells is plotted in Fig. 7b. The value of Δ*E* in this study is defined as the difference between the two potentials at the SOC of 50% in each cycle. The Ti_2_Nb_10_O_27.1_/Li cell exhibits smaller Δ*E* values than those of the Ti_2_Nb_10_O_29_/Li cell at all rates. Especially, the difference between the Δ*E* values in the two cells becomes larger when the rate increases. These results suggest that the defects in Ti_2_Nb_10_O_27.1_ can significantly reduce its polarization and thus can favor its rate performance.

In spite of the slightly smaller capacities at 0.1 C, the Ti_2_Nb_10_O_27.1_/Li cell shows larger capacities at high rates than the Ti_2_Nb_10_O_29_/Li cell. For instance, while the Ti_2_Nb_10_O_29_/Li cell can offer a charge capacity of 185 mAh g^–1^ at 2 C, the Ti_2_Nb_10_O_27.1_/Li cell is able to deliver 209 mAh g^–1^. With further increase of the rate to 5 C (~2 A g^–1^), the charge capacity of the Ti_2_Nb_10_O_27.1_/Li cell still reaches 180 mAh g^–1^, which is twice of that of the Ti_2_Nb_10_O_29_/Li cell (90 mAh g^–1^) and even exceeds the theoretical capacity of the popular Li_4_Ti_5_O_12_/Li cell (175 mAh g^–1^). During the electrochemical reaction of an LIB, electrons and Li^+^ ions simultaneously transport in active material particles. Since both samples in this work have similar particle sizes, their rate performances are determined by their electronic conductivities and Li^+^ ion diffusion coefficients. As demonstrated previously, the defective Ti_2_Nb_10_O_27.1_ exhibits improved electronic conductivity and Li^+^ ion diffusion coefficient, which are at least six orders of magnitude and ~2.5 times larger than those of the stoichiometric Ti_2_Nb_10_O_29_, respectively. These two improvements can facilitate the transport of electrons and Li^+^ ions in the Ti_2_Nb_10_O_27.1_ particles, resulting in the better rate performance and thus higher power density. All these discharge–charge results are well consistent with the previous CV analysis.

### Cyclability

**Figure 8:**
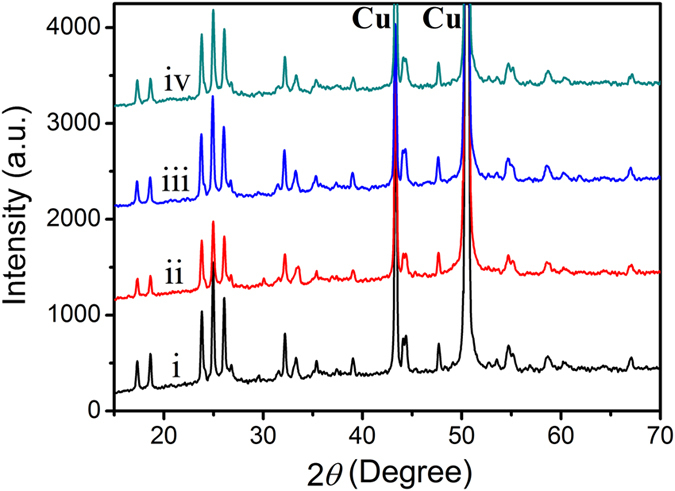
*Ex situ* XRD patterns of Ti_2_Nb_10_O_27.1_ electrodes after (i) as-fabricated, (ii) first-discharged to 0.8 V *vs.* Li/Li^+^, (iii) first-charged to 3 V *vs.* Li/Li^+^, and (iv) charged to 3 V *vs.* Li/Li^+^ in the 10^th^ cycle. Identical discharge–charge rates were used.

The two cells were further subjected to cyclability evaluation at 5 C, as displayed in Fig. 7c. The Ti_2_Nb_10_O_27.1_/Li cell remains a large charge capacity of 164 mAh g^–1^ after 100 cycles, which keeps 91.0% of its initial charge capacity. In sharp contrast, the corresponding values for the Ti_2_Nb_10_O_29_/Li cell are only 67 mAh g^–1^ and 74.7%. Besides the good cyclability, the Ti_2_Nb_10_O_27.1_/Li cell also exhibits excellent Coulombic efficiency of ~100% throughout the cycling (Fig. 7c). These results demonstrate the highly reversible characteristic, outstanding structural stability as well as fast electronic and ionic transport in the Ti_2_Nb_10_O_27.1_ electrode. Its good cyclability is further supported by its *ex situ* XRD result. Figure 8 exhibits the XRD patterns of the Ti_2_Nb_10_O_27.1_ electrodes after as-fabricated, first-discharged to 0.8 V *vs.* Li/Li^+^, first-charged to 3 V *vs.* Li/Li^+^, and charged to 3 V *vs.* Li/Li^+^ in the 10^th^ cycle. As can be seen, the four patterns are very similar. There are little diffraction peak shift and no new peaks at different SOC in spite of some variations in peak intensity, which confirms that basic monoclinic crystal structure of Ti_2_Nb_10_O_27.1_ was maintained during the repeated discharge and charge processes. Therefore, Ti_2_Nb_10_O_27.1_ is an intercalation-type anode material, similar to Ti_2_Nb_10_O_29_ (see Supplementary Fig. S1 online), TiNb_2_O_7_ and Li_4_Ti_5_O_12_. The desirable intercalation/deintercalation characteristic and good structural reversibility of Ti_2_Nb_10_O_27.1_ can also be ascribed to its good cyclability.

In summary, the defective Ti_2_Nb_10_O_27.1_ has been fabricated through a facile solid-state reaction in argon. It shows a Wadsley-Roth shear structure with *A2/m* space group, the same as that of the stoichiometric Ti_2_Nb_10_O_29_. In comparison with Ti_2_Nb_10_O_29_, Ti_2_Nb_10_O_27.1_ not only exhibits larger lattice parameters and a larger unit cell volume but also contains Ti^3+^ ions, Nb^4+^ ions and 6.6% O^2–^ vacancies (*vs.* all O^2–^ ions). As a result of this advanced crystal structure, Ti_2_Nb_10_O_27.1_ has improved electronic conductivity (1.06 × 10^–3^ S cm^–1^) and Li^+^ ion diffusion coefficients (averagely 2.11 × 10^–14^ cm^2^ s^–1^), which are respectively at least six orders of magnitude and ~2.5 times larger than those of Ti_2_Nb_10_O_29_. Consequently, Ti_2_Nb_10_O_27.1_ presents outstanding electrochemical performances in terms of the capacity, rate performance and cyclability. At 0.1 C, it delivers a large initial discharge capacity of 329 mAh g^–1^ and charge capacity of 286 mAh g^–1^. At 5 C, it still remains a large charge capacity of 180 mAh g^–1^ with large capacity retention of 91.0% over 100 cycles, in sharp contrast to the corresponding values of only 90 mAh g^–1^ and 74.7% from Ti_2_Nb_10_O_29_. Clearly, this intercalation-type Ti_2_Nb_10_O_27.1_ possesses the same advantages of Li_4_Ti_5_O_12_ but significantly larger capacities. Therefore, it is able to fulfil the two requirements of high power density and high energy density and thus may be a superior and practical anode material for the LIBs of EVs.

## Method

### Material preparations

The defective Ti_2_Nb_10_O_29–*x*_ was fabricated through a one-step solid-state reaction using TiO_2_ (Sigma–Aldrich, 99.9%) and Nb_2_O_5_ (Sigma–Aldrich, 99.9%) with a predetermined molar ratio of TiO_2_ : Nb_2_O_5_ = 2 : 5. These precursors were mixed and milled by a ball-milling machine (SPEX 8000M) for 4 h, and finally calcined at 1200 °C for 4 h in a tube furnace in an argon atmosphere. As a comparison, the stoichiometric Ti_2_Nb_10_O_29_ was also synthesized by the same process except for the calcination in an air atmosphere.

To prepare the Ti_2_Nb_10_O_29–*x*_ and Ti_2_Nb_10_O_29_ samples for electronic conductivity measurements, the above precursors were uni-axially pressed into pellets with a diameter of 10.25 mm at a pressure of 1000 kg cm^–2^. The pressed pellets were calcined at 850 °C for 5 h and then at 1200 °C for 48 h in argon (for Ti_2_Nb_10_O_29–*x*_) or air (for Ti_2_Nb_10_O_29_). After polishing the two sides of the calcined pellets, gold films were evaporated onto both sides, forming Au/Ti_2_Nb_10_O_29–*x*_/Au and Au/Ti_2_Nb_10_O_29_/Au symmetric ion blocking cells.

### Materials characterizations

Detailed crystal structures of Ti_2_Nb_10_O_29–*x*_ and Ti_2_Nb_10_O_29_ were identified using X-ray diffractions (XRD) combined with Rietveld refinements. XRD patterns for the refinements were recorded in an angle interval of 5–130° (2*θ*) with a step width of 0.03° and a counting time of 8 s per step using an X-ray diffractometer (Bruker D8, Germany) with a monochromatic Cu Kα radiation (λ = 0.1506 nm). *Ex situ* XRD patterns were collected between 15° to 70° (2*θ*) at a scanning speed of 1° min^–1^. The refinements were carried out using the GSAS program with the EXPGUI interface^23^,^24^. During these refinements, the following instrumental and structural parameters were refined: background parameters, zero-shift, unit cell parameters, profile parameters, atomic fractional coordinates, atomic isotropic displacement parameters and atomic occupancies. The site occupancies were constrained to the designed chemical formulas. Morphologies, particle sizes and microstructures were examined using a field emission scanning electron microscopy (FESEM, Hitachi S-4800, Japan) and a high-resolution transmission electron microscopy (HRTEM, JEOL JEM-2100, Japan). Nitrogen adsorption–desorption isotherms at 77 K were obtained in a surface area analyser (Quantachrome NOVA 2200e, USA). Specific surface areas were derived based on the Brunauer–Emmett–Teller (BET) model. Electronic conductivity tests were performed on the ion blocking cells using an electrochemical workstation (Zahner zennium, Kronach, Germany) under a small voltage of 50 mV until the corresponding currents stabilized.

### Electrochemical tests

Electrochemical performances were examined by the tests of CR2016 coin cells assembled in an argon-filled glove box (Mbraum, Unilab, Germany). In these cells, pure Li foils were used as counter and reference electrodes, microporous polypropylene films (Celgard 2400, Celgard LLC., USA) as separators, and a mixture of ethylene carbonate, dimethyl carbonate and diethylene carbonate (1 : 1 : 1 by weight) containing 1 M LiPF_6_ (DAN VEC) as electrolyte. Their corresponding working electrodes were fabricated by a slurry-coating procedure. The slurries contained 65 wt% active materials (Ti_2_Nb_10_O_29–*x*_ or Ti_2_Nb_10_O_29_), 25 wt% super P^®^ conductive carbon (TIMCAL Ltd.) and 10 wt% polyvinylidene fluoride (PVDF, Sigma–Aldrich) in N-methylpyrrolidone (NMP, Sigma–Aldrich). After homogeneously blended, these slurries were uniformly coated on Cu plates. The coated plates were then dried in a vacuum oven at 120 °C for 10 h and finally roller-pressed by a rolling machine to form the working electrodes.

Galvanostatic discharge–charge tests were conducted using a multi-channel battery testing system (LANHE CT2001A, China) with a cut-off potential of 3.0–0.8 V *vs.* Li/Li^+^. All discharge/charge rates were denoted using C-rate where 396 mA g^–1^ was assigned to the current density of 1 C based on the theoretical capacity of Ti_2_Nb_10_O_29_ (396 mAh g^–1^). To prepare the electrodes for the *ex situ* XRD experiments, the coin cells at different states of charge (SOC) were disassembled, and then the working electrodes were washed by dimethyl carbonate for three times and dried at 80 °C. Cyclic voltammetry (CV) measurements were performed using the above electrochemical workstation.

### Calculation methodology

All calculations were performed using the projector-augmented wave (PAW) method within the density functional theory (DFT), as implemented in the Vienna *ab initio* simulation package (VASP)^25^,^26^,^27^. Electronic exchange-correlation functional was treated within the spin-polarized generalized gradient approximation (GGA) parameterized by Perdew-Burke-Ernzerhof (PBE)^28^. To address the on-site Coulombic interactions in the localized *d* electrons of Nb ions, the GGA + *U* method with an additional Hubbard-type *U* term (*U*_eff_ = *U* – *J* = 1.5 eV) was employed, which has been proved to be a good approximation for Nb in electrode materials of LIBs^29^,^30^. Wave functions are expanded in plane waves up to a kinetic energy cut-off of 500 eV. Brillouin-zone integrations were approximated using special k-point sampling of Monkhorst-Pack scheme^31^ with a k-point mesh resolution of 2π × 0.05 Å^−1^. Lattice vectors (both unit cell shape and size) are fully relaxed together with atomic coordinates until the Hellmann-Feynman force on each atom is less than 0.01 eV/Å.

## Additional Information

**How to cite this article**: Lin, C. *et al.* Defective Ti_2_Nb_10_O_27.1_: an advanced anode material for lithium-ion batteries. *Sci. Rep.* **5**, 17836; doi: 10.1038/srep17836 (2015).

## Supplementary Material

Supplementary Information

## Figures and Tables

**Table 1:** Results of crystal analysis by Rietveld refinements in Ti_2_Nb_10_O_29_ and Ti_2_Nb_10_O_27.1_ (^*a*^Occupancy of O^2–^ in O3 sites. ^*b*^Occupancy of O^2–^ in O4 sites.

Sample	*a* (Å)	*b* (Å)	*c* (Å)	*α*, *γ* (˚)	*β* (˚)	*V* (Å^3^)	*f*_1_^*a*^	*f*_2_^*b*^	*f*_3_^*c*^	*R*_wp_^*d*^	*R*_p_^*e*^	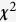 ^*f*^
Ti_2_Nb_10_O_29_	15.52368 (58)	3.81104 (11)	20.54769 (67)	90 (–)	113.058 (3)	1118.512 (78)	1 (–)	1 (–)	1 (–)	0.0998	0.0789	6.593
Ti_2_Nb_10_O_27.1_	15.53722(67)	3.81393 (13)	20.55602 (78)	90 (–)	113.070 (3)	1120.694 (92)	0.339 (31)	0.710 (32)	1 (–)	0.0940	0.0733	5.638

^*c*^Occupancy of O^2–^ in other O sites. ^*d*^Weighted profile residual. ^*e*^Profile residual. ^*f*^Goodness of fit).

## References

[b1] Armand M. & Tarascon J. M. Building better batteries. Nature 154, 652–657 (2008). 18256660 10.1038/451652a

[b2] Zheng S. S. The effect of the charging protocol on the cycle life of a Li-ion battery. J. Power Sources 161, 1385–1391 (2006).

[b3] Goodenough J. B. & Kim Y. Challenges for Rechargeable Li Batteries. Chem. Mater. 22, 587–603 (2010).

[b4] Ohzuku T., Ueda A. & Yamamoto N. Zero-Strain Insertion Material of Li[Li_1/3_Ti_5/3_]O_4_ for Rechargeable Lithium Cells. J. Electrochem. Soc. 142, 1431–1435 (1995).

[b5] Lin C. F. *et al.* Advanced electrochemical performance of Li_4_Ti_5_O_12_-based materials for lithium-ion battery: Synergistic effect of doping and compositing. J. Power Sources 248, 1034–1041 (2014).

[b6] Lin C. F. *et al.* Li4Ti5O12-based anode materials with low working potentials, high rate capabilities and high cyclability for high-power lithium-ion batteries: a synergistic effect of doping, incorporating a conductive phase and reducing particle size. J. Mater. Chem. A 2, 9982–9993 (2014).

[b7] Han J. T., Huang Y. H. & Goodenough J. B. New Anode Framework for Rechargeable Lithium Batteries. Chem. Mater. 23, 2027–2029 (2011).

[b8] Han J. T. & Goodenough J. B. 3-V Full Cell Performance of Anode Framework TiNb2O7/Spinel LiNi0.5Mn1.5O4. Chem. Mater. 23, 3404–3407 (2011).

[b9] Wu X. Y. *et al.* Investigation on Ti2Nb10O29 anode material for lithium-ion batteries. Electrochem. Commun. 25, 39–42 (2012).

[b10] Cava R. J., Murphy D. W. & Zahurak S. M. Lithium Insertion in Wadsley–Roth Phases Based on Niobium Oxide. J. Electrochem. Soc. 130, 2345–2351 (1983).

[b11] Fei L. *et al.* SBA-15 confined synthesis of TiNb_2_O_7_ nanoparticles for lithium-ion batteries, Nanoscale 5, 11102–11107 (2013). 24071825 10.1039/c3nr03594h

[b12] Jayaraman S. *et al.* Exceptional Performance of TiNb_2_O_7_ Anode in All One-Dimensional Architecture by Electrospinning. ACS Appl. Mater. Interfaces 6, 8660–8666 (2014). 24766070 10.1021/am501464d

[b13] Tang K., Mu X. K., van Aken P. A., Yu Y. & Maier J. “Nano-Pearl-String” TiNb2O7 as Anodes for Rechargeable Lithium Batteries, Adv. Energy Mater. 3, 49–53 (2013).

[b14] Jo C. *et al.* Block Copolymer Directed Ordered Mesostructured TiNb2O7 Multimetallic Oxide Constructed of Nanocrystals as High Power Li-Ion Battery Anodes. Chem. Mater. 26, 3508–3514 (2014).

[b15] Guo B. K. *et al.* A long-life lithium-ion battery with highly porous TiNb2O7 anode for large-scale electrical energy storage. Energy Environ. Sci. 7, 2220–2226 (2014).

[b16] Cheng Q. S. *et al.* Bulk Ti_2_Nb_10_O_29_ as long-life and high-power Li-ion battery anodes. J. Mater. Chem. A 2, 17258–17262 (2014).

[b17] Lu X. *et al.* Atomic-scale investigation on lithium storage mechanism in TiNb_2_O_7_. Energy Environ. Sci. 4, 2638–2644 (2011).

[b18] Chen X. M., Guan X. F., Li L. P. & Li G. S. Defective mesoporous Li_4_Ti_5_O_12−*y*_: An advanced anode material with anomalous capacity and cycling stability at a high rate of 20 C. J. Power Sources 210, 297–302 (2012).

[b19] Shannon R. D. Revised Effective Ionic Radii and Systematic Studies of Interatomic Distances in Halides and Chalcogenides. Acta Crystallogr., Sect. A: Found. Crystallogr. 32, 751–767 (1976).

[b20] Burns R. G. Mineralogical Applications Of Crystal Field Theory 2nd edn, (Cambridge University Press, 1993).

[b21] Lin C. F., Lai M. O., Lu L. & Zhou H. H. Spinel Li_4_−_2*x*_Co_3*x*_Ti_5_−_*x*_O_12_ (0 ≤ *x* ≤ 0.5) for Lithium-Ion Batteries: Crystal Structures, Material properties, and Battery Performances. J. Phys. Chem. C 118, 14246–14255 (2014).

[b22] Bard A. J. & Faulkner L. R. Electrochemical Methods: Fundamentals And Applications 2nd edn, (Wiley, 2001).

[b23] Larson A. C. & Von Dreele R. B. General Structure Analysis System (GSAS), Los Alamos National Laboratory Report LAUR 86, 748 (1994).

[b24] Toby B. H. EXPGUI, a graphical user interface for GSAS. J. Appl. Cryst. 34, 210–213 (2001).

[b25] Kresse G. & Joubert D. From ultrasoft pseudopotentials to the projector augmented-wave method. Phys. Rev. B: Condens. Matter Mater. Phys. 59, 1758–1775 (1999).

[b26] Kresse G. & Furthmüller J. Efficiency of *ab-initio* total energy calculations for metals and semiconductors using a plane-wave basis set. Comput. Mater. Sci. 6, 15–50 (1996). 10.1103/physrevb.54.11169 9984901

[b27] Kresse G. & Furthmüller J. Efficient iterative schemes for *ab initio* total-energy calculations using a plane-wave basis set. Phys. Rev. B: Condens. Matter 54, 11169–11186 (1996). 9984901 10.1103/physrevb.54.11169

[b28] Perdew J. P., Burke K. & Ernzerhof M. Generalized Gradient Approximation Made Simple. Phys. Rev. Lett. 77, 3865–3868 (1996). 10062328 10.1103/PhysRevLett.77.3865

[b29] Dudarev S. L., Botton G. A., Savrasov S. Y., Humphreys C. J. & Sutton A. P. Electron-energy-loss spectra and the structural stability of nickel oxide: an LSDA + U study. Phys. Rev. B 57, 1505–1509 (1998).

[b30] Mueller T., Hautier G., Jain A. & Ceder G. Evaluation of Tavorite-Structured Cathode Materials for Lithium-Ion Batteries Using High-Throughput Computing. Chem. Mater. 23, 3854–3862 (2011).

[b31] Monkhorst H. J. & Pack J. D. Special points for Brillouin-zone integrations. Phys. Rev. B 13, 5188–5192 (1976).

