# Supplementary material for: Defective Ti2Nb10O27.1: an advanced anode material for lithium-ion batteries
Source: Sci Rep. 2015 Dec 3;5:17836. doi: 10.1038/srep17836 (PMC4668578; doi:10.1038/srep17836)
Supplement: Supplementary Information [file srep17836-s1.pdf]

Supplementary information

Defective $Ti_2Nb_{10}O_{27.1}$: an advanced anode material for lithium-ion batteries of electric vehicles

Chunfu Lin[1,*,‡], Shu Yu[2,‡], Hua Zhao[1], Shunqing Wu[2,*], Guizhen Wang[1], Lei Yu[1], Yanfang Li[1], Zi-Zhong Zhu[2], Jianbao Li[1] & Shiwei Lin[1,*]

[1]Key Laboratory of Ministry of Education for Advanced Materials in Tropical Island Resources, College of Materials and Chemical Engineering, Hainan University, Haikou 570228, PR China.

[2]Department of Physics and Institute of Theoretical Physics and Astrophysics, Xiamen University, Xiamen 361005, PR China.

[‡]These authors contributed equally to this work.

Correspondence and requests for materials should be addressed to C.L. (email: linchunfu@hainu.edu.cn) or S.W. (email: wsq@xmu.edu.cn) or S.L. (email: linsw@hainu.edu.cn).

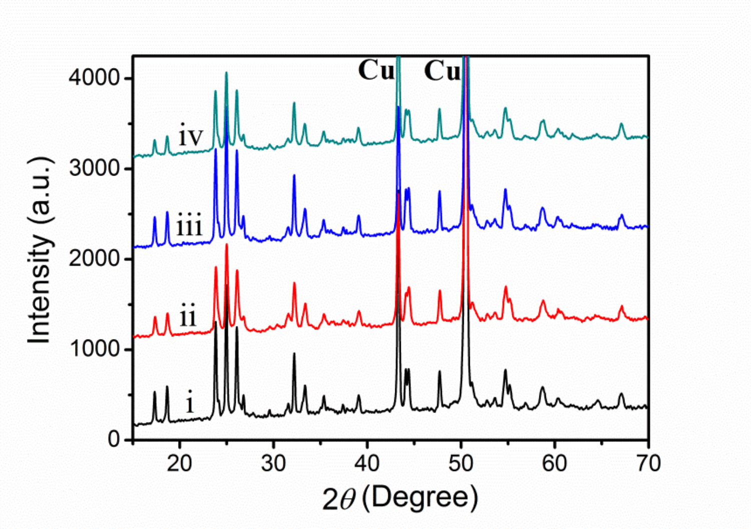

Supplementary Figure S1. *Ex situ* XRD patterns of $Ti_2Nb_{10}O_{29}$ electrodes after (i) as-fabricated, (ii) first-discharged to 0.8 V *vs.* Li/Li^+, (iii) first-charged to 3 V *vs.* Li/Li^+, and (iv) charged to 3 V *vs.* Li/Li^+ in the 10th cycle. Identical discharge–charge rates were used.
